# Low-Rank Plus Sparse Decomposition of fMRI Data With Application to Alzheimer's Disease

**DOI:** 10.3389/fnins.2022.826316

**Published:** 2022-03-14

**Authors:** Wei Tu, Fangfang Fu, Linglong Kong, Bei Jiang, Dana Cobzas, Chao Huang

**Affiliations:** ^1^Canadian Cancer Trials Group and Department of Public Health Sciences, Queen's University, Kingston, ON, Canada; ^2^Department of Mathematical and Statistical Sciences, University of Alberta, Edmonton, AB, Canada; ^3^Department of Computer Science, MacEwan University, Edmonton, AB, Canada; ^4^Department of Statistics, Florida State University, Tallahassee, FL, United States

**Keywords:** functional connectivity, rsfMRI = resting-state fMRI, low rank plus sparse decomposition (LRSD), Alzheimer's disease, ADNI

## Abstract

Studying functional brain connectivity plays an important role in understanding how human brain functions and neuropsychological diseases such as autism, attention-deficit hyperactivity disorder, and Alzheimer's disease (AD). Functional magnetic resonance imaging (fMRI) is one of the most popularly used tool to construct functional brain connectivity. However, the presence of noises and outliers in fMRI blood oxygen level dependent (BOLD) signals might lead to unreliable and unstable results in the construction of connectivity matrix. In this paper, we propose a pipeline that enables us to estimate robust and stable connectivity matrix, which increases the detectability of group differences. In particular, a low-rank plus sparse (*L* + *S*) matrix decomposition technique is adopted to decompose the original signals, where the low-rank matrix *L* recovers the essential common features from regions of interest, and the sparse matrix *S* catches the sparse individual variability and potential outliers. On the basis of decomposed signals, we construct connectivity matrix using the proposed novel concentration inequality-based sparse estimator. In order to facilitate the comparisons, we also consider correlation, partial correlation, and graphical Lasso-based methods. Hypothesis testing is then conducted to detect group differences. The proposed pipeline is applied to rs-fMRI data in Alzheimer's disease neuroimaging initiative to detect AD-related biomarkers, and we show that the proposed pipeline provides accurate yet more stable results than using the original BOLD signals.

## 1. Introduction

Alzheimer's disease (AD) is a chronic irreversible neurodegenerative disease. It is recognized as a major public health problem, as it eventually affects every aspect of people's life (MacDonald et al., 2015). AD usually progresses very slowly and gradually worsen over a number of years, becoming serious enough to interfere with people's daily life (ADs, 2022). Scientists have found that many diseases are associated with changes in brain connectivity, such as autism, attention-deficit hyperactivity disorder (ADHD), and AD (Konrad and Eickhoff, 2010; Uddin et al., 2013; Dennis and Thompson, 2014). Also, it was shown that individual's functional brain connectivity can act as an identifying fingerprint, which is unique, intrinsic, and reliable (Finn et al., 2015). Hence, studying functional brain connectivity is of great importance to better understand the mechanisms of AD. Functional brain connectivity is defined as the correlations between measurements of neuronal activity in different brain areas (Friston, 2011). In practice, it can be evaluated by functional neuroimaging (Van Den Heuvel and Pol, 2010). Among different imaging modalities, functional magnetic resonance imaging (fMRI) measures brain activity by detecting changes associated with blood flow. The primary form of fMRI uses the blood oxygen level dependent (BOLD) contrast.

Due to scanner instabilities, acquisition, or issues in the underlying biomedical experimental protocol, however, fMRI BOLD signals might contain noise and outliers (Magnotti and Billor, 2014). The presence of potential outliers in the fMRI signals might lead to unreliable and unstable results in the constructed connectivity matrices. In order to deal with these challenges, we propose a framework that enables us to provide robust and stable functional connectivity matrices, which can further increase the detectability of group differences. In particular, a low-rank plus sparse (*L* + *S*) matrix decomposition technique is adapted to decompose the fMRI BOLD signals, where the low-rank matrix *L* recovers the essential common features between regions of interest (ROIs), and the sparse matrix *S* catches the sparse individual variability and potential outliers (Baete et al., 2018). There are some existing algorithms that can solve this problem computationally, such as Accelerated Proximal Gradient (APG), Augmented Direction Method (ADM), Augmented Lagrange Multiplier (ALM), and so on. Bouwmans and Zahzah (2014).

After the *L* + *S* matrix decomposition of original fMRI signals, we use different methods to construct the functional brain connectivity matrix. Various methods have been developed in the literature, such as correlation, partial correlation, graphical Lasso (GLasso), and so on. Since the fMRI data have high dimensionality (especially at voxel level), the classical asymptotic correlation estimation might perform poorly (Kashlak and Kong, 2017). In this paper, we propose to use the recently developed novel non-asymptotic sparse matrix estimation based on concentration inequality to construct the functional connectivity, which is shown to have superior performance than other methods. Once we build the functional connectivity matrices for both the normal control group and disease group, we can conduct the comparisons in order to identify the group differences, which is essential for uncovering underlying neurological processes associated with the corresponding disease. Since functional connectivity is not directly observed, but has to be estimated from noisy and complex imaging data. The comparison of the functional connectivity at the group level highly depends on many factors such as the estimation of the connectivity matrices, the models used in the testing or comparison and other practical issues related to the application. There have been many work in the literature addressing these challenges, for example, Kim et al. (2015) compared the use of correlation and partial correlation; (Narayan and Allen, 2016) proposed the use of mixed effects model to account for other covariate effects; (Wozniak et al., 2013) presented an application comparing global functional connectivity abnormalities in children with fetal alcohol spectrum disorders. Detecting group differences for specific diseases associated with functional connectivity is critical for both research and clinical uses.

**Figure 1:**
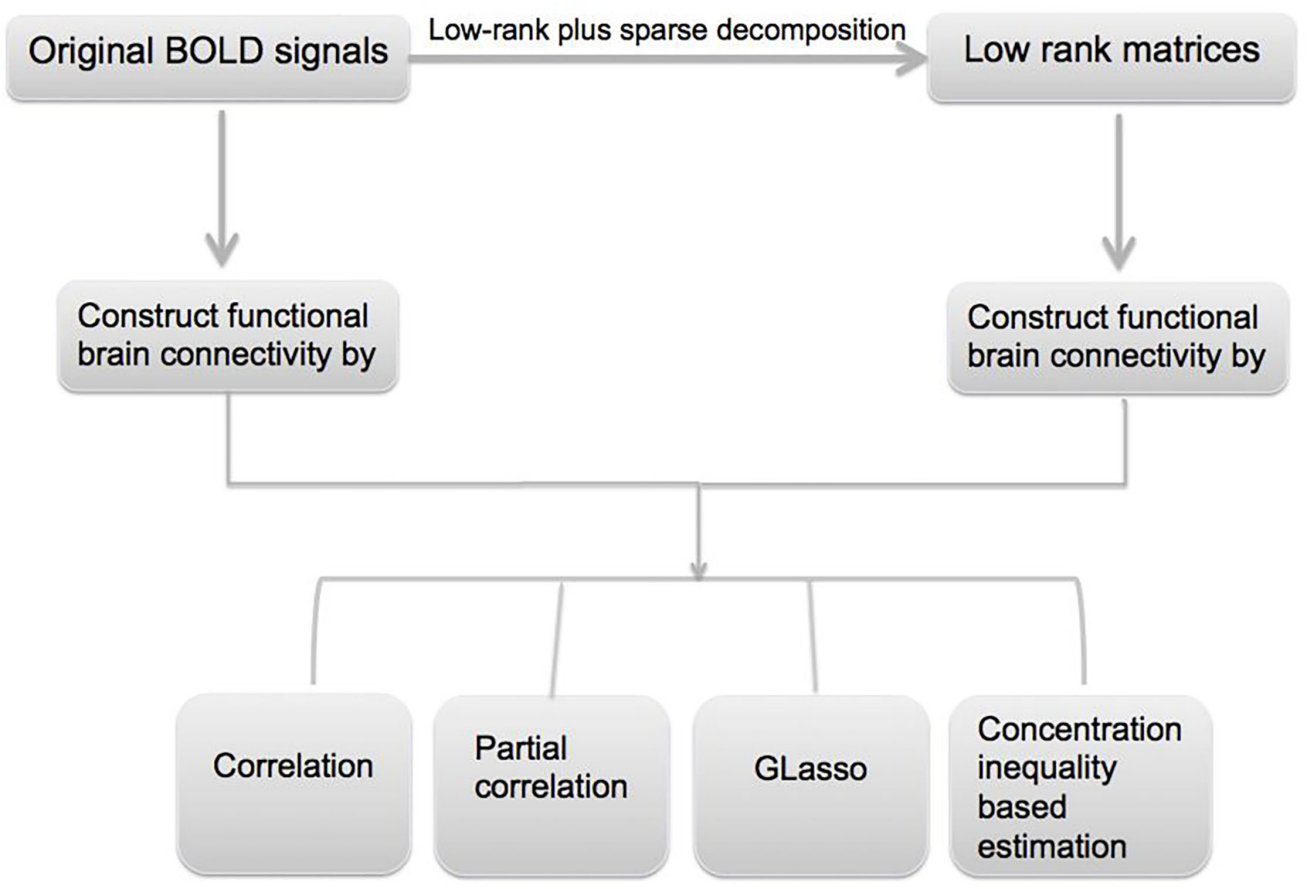
Flowchart for the proposed robust functional brain connectivity pipeline.

In this paper, we utilize resting-state fMRI data from Alzheimer's Disease Neuroimaging Initiative (ADNI) database (www.loni.ucla.edu/ADNI). The overall goal of the data analysis is to identify functional connectivity biomarkers for AD, and it mainly involves three steps. First, an *L* + *S* decomposition is applied to the original fMRI signals to obtain the common features in the data; second, the obtained low-rank signal is used to construct the functional connectivity matrices, and different methods such as Glasso and concentration inequality-based estimation have been applied; lastly, hypothesis testing has been conducted on the estimated functional connectivity matrices to detect biomarkers related to AD. A flowchart of the data analysis can be found in Figure 1.

The rest of the paper is organized as follows. In Section 2, we introduce some technical details on low rank plus sparse matrix decomposition. In Section 3, we present the proposed novel concentration inequality-based sparse matrix estimation. To facilitate the comparisons, we also introduce correlation, partial correlation, and Graphical Lasso-based correlation matrix estimation. In Section 4, we apply our method to the ADNI data and illustrate the advantages of the proposed pipeline. Conclusions and potential future work can be discussed in Section 5.

## 2. Low-rank Plus Sparse Matrix Decompositions

*L* + *S* matrix decomposition is a special type of matrix decomposition. It originates from robust principal component analysis. Principal component analysis (PCA) tries to find a low subspace that approximates the original data matrix by exploring the eigen-structure of the correlation matrix. However, the original PCA is sensitive to outliers. To improve the robustness performance of PCA, researchers have developed various robust PCA methods (Jolliffe and Cadima, 2016). Early attempts to solve the RPCA problem have been conducted (Xu and Yuille, 1995; Croux and Haesbroeck, 2000; De la Torre and Black, 2001; De La Torre and Black, 2003; Croux and Ruiz-Gazen, 2005). However, they could not achieve polynomial time solutions with high performance. A more recent version of RPCA becomes increasingly popular (Kang et al., 2015).

The straightforward formulation for RPCA is to employ *l*_0_-norm to solve


$$\begin{array}{l} \underset{L,S}{\min}\text{rank}\left(L\right)+\lambda {\left\|S\right\|}_{0},\text{ s.t. }L+S=M, \end{array}$$

where *M* is a *m* × *n* matrix to be decomposed, *L* is a low-rank matrix, *S* is a sparse matrix, ∥·∥_0_ is the *l*_0_-norm, and λ is a non-negative tuning parameter. But this optimization problem is not convex and NP-hard. Hence, it was suggested in Candès et al. (2011) that we can approximate this problem by a convex optimization problem, which is to minimize a combination of the *l*1 norm of *S* and the nuclear norm of *L* (Wright et al., 2009). This essentially is the *L* + *S* matrix decomposition. Specifically, the *L* + *S* matrix decomposition can be expressed as


(1) $$\begin{array}{l} \underset{L,S}{\min}{\left\|L\right\|}_{*}+\lambda {\left\|S\right\|}_{1},\text{ s.t. }L+S=M, \end{array}$$

where ∥·∥_*_ is the nuclear norm (sum of all singular values), ∥·∥_1_ is the *l*1-norm, and λ is a non-negative tuning parameter. Smaller λ can enforce lower rank for *L*, but relax the sparsity for *S*.

There are some existing algorithms that can solve this problem computationally, such as APG, ADM, ALM, and so on Bouwmans and Zahzah (2014). In this paper, the algorithm ALM was chosen because it is much faster, achieve higher precision and being less storage/memory demanding comparing with other popular choices such as APG (Lin et al., 2010). The R package for ALM is available online.

In this paper, we apply the *L* + *S* matrix decomposition to the fMRI BOLD signals. More specifically, let *X*_*N*×*T*×*J*_ = {*x*_*itj*_} be a 3-dimensional tensor where *i* represents the *i*th subject (*i* = 1, …, *N*), *t* represents the *t*th time course (*t* = 1, …, *T*), and *j* represents *j*th ROI (*j* = 1, …, *J*). Then for each fixed ROI, we conduct *L* + *S* matrix decomposition to each *X*_*N*×*T*_, and we can obtain a low-rank new 3-dimensional matrix denoted as *L*_*N*×*T*×*J*_ = *l*_*itj*_, which represents the common features among the fMRI signals across the subjects. Then, we built functional brain connectivity-based on each *L*_*T*×*J*_. Here, the original fMRI signals *X*_*N*×*T*_ is equivalent to the *M* matrix in equation (1), and the resulting low-rank *L*_*T*×*J*_ is equivalent to the *L* matrix.

## 3. Concentration Inequality- Based Estimation of Sparse Covariance Matrices

Estimation of correlation and covariance matrices using high-dimensional data is an important topic. Many estimators of covariance matrix have been explored working under the assumption of sparsity, which is desirable and applicable in high dimensional settings since many variable pairings might be considered uncorrelated. Shrinkage estimators, and thresholding estimators, for example, are two classes for sparse estimators of covariance matrix (Kashlak and Kong, 2017).

Let vectors $X_{1},\cdots X_{T}\in {\mathbb{R}}^{J}$ be a sample with mean zero and covariance matrix Σ, and *S* is the sample covariance of Σ for each subject. When the dimension *J* is large and Σ is sparse, *S* might not be a good estimator. To construct a better estimator, a novel approach is recently proposed making use of confidence sets constructed from concentration inequalities for non-asymptotic covariance matrix estimation. Let ${\hat{\Sigma }}^{sp}$ be the sparse estimator for Σ. This method chooses a ${\hat{\Sigma }}^{sp}$ such that it is close enough to *S* while it lies far enough away to result in a sparse estimator. This novel concentration inequality-based method supplies finite sample guarantees and a much faster computing time compared with costly optimization and cross-validation methods (Kashlak and Kong, 2017).

The sparse estimation procedure aims at constructing a sparse estimator ${\hat{\Sigma }}^{sp}$ for Σ by constructing a non-asymptotic confidence set first employing concentration inequalities for Σ based on *S*, and searching this set in order to obtain the sparsest estimator. The general form for the concentration inequalities is


$$P(d(\Sigma ,S)\geq Ed(\Sigma ,S)+r)\leq e^{-\psi (r)},$$

where ψ:ℝ → ℝ is a monotonically increasing function, and *d*(·) is some metric measuring the distance of two covariance matrices. To construct a 1 − α confidence set, *r* = *r*_α_ is chosen such that exp(−ψ(*r*_α_)) = α. The smaller α is, the larger *r* is. Our sparse estimator ${\hat{\Sigma }}^{sp}$ is expected to be close to Σ in the sense of the above confidence set, and thus we focus on choosing a ${\hat{\Sigma }}^{sp}$ such that $d\left({\hat{\Sigma }}^{sp},S\right)\leq r_{\alpha }$. It begins with *S* and attempts to threshold it as much as possible while still remaining in the confidence set. We define a generalized thresholding operator (Rothman et al., 2009) as *s*_λ_(·):ℝ → ℝ such that


$$|s_{\lambda }(z)|\leq z,s_{\lambda }(z)=0\text{ for }|z|\leq \lambda ,\text{ and }|s_{\lambda }(z)-z|\leq \lambda$$

which will apply to each single element of a matrix. In the past, this estimator was applied to *S* for some λ chosen by cross-validation. Instead of choosing the threshold λ, this approach tries to choose a confidence level 1 − α and then seek for the largest λ such that *d*(*s*_λ_(*S*), *S*) ≤ *r*_α_ (Kashlak and Kong, 2017).

The algorithm for how to derive the sparse covariance matrix estimation is shown in Algorithm 2 (Kashlak and Kong, 2017).

**Algorithm 1:** Concentration inequality-based estimation of sparse covariance matrices

0. Set ${\hat{\Sigma }}_{0}^{sp}={\left({\hat{\Sigma }}^{diag}\right)}^{-1/2}S{\left({\hat{\Sigma }}^{diag}\right)}^{-1/2}$, λ = 0.5 and write ${\hat{\Sigma }}_{\lambda }^{sp}=s_{\lambda }\left(S\right)$. Define *k* = 1 as the number of the recursion's steps. Choose an α and compute *r*_α_.
1. Increase *k*←*k* + 1, then update the threshold λ as below:
**if** $d\left({\hat{\Sigma }}_{\lambda }^{sp}-S\right)\leq r_{\alpha }$, **then**
let λ←λ + 2^−*k*−1^.
**else**
let λ←λ − 2^−*k*−1^.
**end if**
2. Repeat step 1 until *k* has gotten to the desired number of iterations. Generally, as few as *k* = 10 will suffice.
3. The resulting sparse estimator is ${\hat{\Sigma }}^{sp}={\left({\hat{\Sigma }}^{diag}\right)}^{1/2}\left(\hat{\Sigma_{\lambda }^{sp}}\right){\left({\hat{\Sigma }}^{diag}\right)}^{1/2}$, where ${\hat{\Sigma }}_{\lambda }^{sp}$ is our final sparse estimator.

In our study, we employ the operator norm $∥{\hat{\Sigma }}_{\lambda }^{sp}-S{∥}_{\infty }$ as the distance metric *d*(·, ·). We choose reasonable false positive rate α in order to get the reasonable sparsity for the estimation of sparse covariance matrices. Once we obtain the sparse covariance estimation, we can calculate the corresponding correlation matrix as functional brain connectivity.

To facilitate the comparisons, we also employ correlation, partial correlation, and Graphical Lasso methods for constructing the connectivity matrices in our study. The Pearson's correlation between ROIs can be calculated based on a sample covariance matrix. Specifically, let the matrix *M*_*J*×*T*_ be the *i*th subject's BOLD signals or low-rank matrix from decomposition, and each column $M_{1},\cdots M_{T}\in {\mathbb{R}}^{J}$. Then the sample covariance matrix can be derived from $S=s_{pq}={\left(T-1\right)}^{-1}\sum_{i=1}^{T}\left(M_{i}-\bar{M}\right){\left(M_{i}-\bar{M}\right)}^{T}$, where $\bar{M}=T^{-1}\sum_{i=1}^{T}M_{i}$. Then the full correlation between the *p*th ROI and the *q*th ROI can be estimated as $r_{pq}=s_{pq}/{\left(s_{pp}s_{qq}\right)}^{\frac{1}{2}}$. When estimating partial correlations, a precision matrix (or inverse covariance matrix) can be used (Kim et al., 2015). Define the precision matrix $\Theta_{pq}=\left(\theta_{pq}\right)=\Sigma^{-1}$, where Σ is covariance matrix, then the partial correlation between the *p*th ROI and the *q*th ROI is $\rho_{pq}=-\theta_{pq}/{\left(\theta_{pp}\theta_{qq}\right)}^{\frac{1}{2}}$. If the number of ROIs is relatively large, then our derived correlation and partial correlation matrices would be also relatively with high dimensions. In practice, the fact is for the most of time we might only be interested in selecting those connection pairs with larger correlation coefficient values, which means they have stronger connections compared to the others. In order to achieve this goal, we apply thresholding values to both correlation and partial correlation matrices (Cai and Liu, 2011; Fan et al., 2016). In this way, we would get sparse correlation and partial correlation matrices, which would help us focusing on the relatively more important connections among those ROIs. Specifically, let *R* = (_*r*_*ij*_)*J*×*J*_ be the sample correlation matrix, and let τ be the reasonable pre-specified thresholding value. Then we enforce the thresholding value to all the off diagonal elements of our correlation matrix to get the corresponding sparse correlation matrix *R*^*sp*^, i.e., for the (*i, j*)th element of *R*^*sp*^,


$$R_{\left(i,j\right)}^{sp}=\{\begin{array}{cc} 1, & i=j\\ r_{ij}1\left\{|r_{ij}|>\tau \right\}, & i\neq j \end{array}.$$

When τ = 1, it is an identity matrix, while when 0, it is the original sample correlation matrix as we do not apply any thresholding. The same thresholding method is also applied to the estimated partial correlation matrices in the paper. The Graphical Lasso problem is to maximize the *l*1-penalized Gaussian log-likelihood


(2) $$\begin{array}{l} \log\left(\det\Theta \right)-\mathrm{tr}\left(S\Theta \right)-\rho ∥\Theta {∥}_{1}, \end{array}$$

over non-negative definite matrices Θ, where Θ = Σ^−1^, tr denotes the trace, *S* is the sample covariance matrix, ρ is a non-negative tuning parameter, and ∥Θ∥_1_ is the *l*_1_ norm, the sum of the absolute values of all the entries of Σ^−1^ (Friedman et al., 2008). When ρ is 0, then there is no penalty. When ρ is sufficiently large, the estimate $\hat{\Theta }$ will be sparse due to the Lasso-type penalty. In our study, the value ρ in (2) is chosen to get the reasonable sparsity. The problem (2) is shown to be convex (Banerjee et al., 2008).

## 4. Detecting Group Differences

In our study, in order to detect group differences in functional brain connectivity, we conduct two sample *t* statistical test to test whether there is any difference for each single ROI connection between the control group and disease group. Theoretically, if we have *J* ROIs, then we have *J*(*J* − 1)/2 total connections. Suppose a connectivity matrix $C_{J\times J}^{\left(i\right)}$ for *i*th subject can be denoted as $\left(c_{p,q}^{\left(i\right)}\right)$, where $c_{p,q}^{\left(i\right)}$ represents the connectivity metric of the *p*th ROI and *q*th ROI, *p* = 1, 2, ⋯ , *J*, and *q* = 1, 2, ⋯ , *J* (e.g., for correlation method, it is the correlation coefficient). Then for each single ROI pair $c_{p,q}^{\left(i\right)}$ of matrix $C_{J\times J}^{\left(i\right)}$, we will have one group of values: $c_{p,q}^{\left(1\right)},c_{p,q}^{\left(2\right)},\cdots c_{p,q}^{\left(N_{1}\right)}$ from the control group and another group of values: $c_{p,q}^{\left(1\right)},c_{p,q}^{\left(2\right)},\cdots c_{p,q}^{\left(N_{2}\right)}$ from the disease group. Then we can conduct two sample *t*-test for each single connection *c*_*p,q*_ of *p*th ROI and *q*th ROI to test whether there is any difference for each single connection between the control group and disease group. Afterward, we can generate a *p*-value matrix *P*_*J*×*J*_ = (*p*_*p,q*_), where *p*_*p,q*_ is the corresponding *p*-value indicating the significance among brain connectivity *c*_*p,q*_ between the control group and disease group. Benjamin–Hochberg procedure is applied to control the false discovery rate.

## 5. Application and Results

### 5.1. Data Description

The ADNI ^1^ is a global longitudinal study for AD through the enrollment and follow-up of cohorts of individuals who have mild cognitive impairment (MCI) and mild AD. The study is designed for the detection at the earliest possible stage and tracking the progression of AD with biomarkers to assess the brain structure and the brain function. The participants enrolled by ADNI are between 55 and 90 years of age, selected based on the particular criteria, and recruited at the 57 ADNI acquisition sites located in the United States and Canada. The five cohorts in this study are normal control (NC), significant memory concern (SMC), early mild cognitive impairment (EMCI), late mild cognitive impairment (LMCI), and AD.

The dataset used in the study contains the NYU site (New York University Child Study Center) with 222 subjects. These 222 subjects include 5 disease categories depending on the severity of AD: 0 (NC), 1 (SMC), 2 (EMCI), 3 (LMCI), and 4 (AD). Since the final comparison of the proposed method involves only a two sample hypothesis testing problem, the most two extreme groups (normal control (*n* = 33) and AD (*n* = 24)) were used. The fMRI data are preprocessed using Automated Anatomical Labeling (AAL) template (Tzourio-Mazoyer et al., 2002). The non-overlapping ROIs are then extracted for each subject. For each subject, each time-series and ROI are computed through averaging all the voxels' time series within the ROIs (Sanz-Arigita et al., 2010). Hence, each subject has BOLD signal data at 116 ROIs through 134 equally spaced time courses. All subjects had 1.5 Tesla and 3 Tesla scans by Philips scanners, having their eyes open when receiving the scanning (SCA, 2017).

### 5.2. ADNI Data Results

We conduct *L* + *S* decomposition to the original BOLD signals for each ROI. Specifically using cross-validation λ = 0.086 is chosen and the rank of the matrices *L*_*N*×*T*_ is reduced to around 30, approximately half as much as the original rank 57 of the decomposed matrix *X*_*N*×*T*_. Then we construct functional connectivity matrices using correlation, partial correlation, Graphical Lasso, and the concentration inequality-based estimation method based on both original BOLD signals and low-rank matrices, respectively.

**Figure 2:**
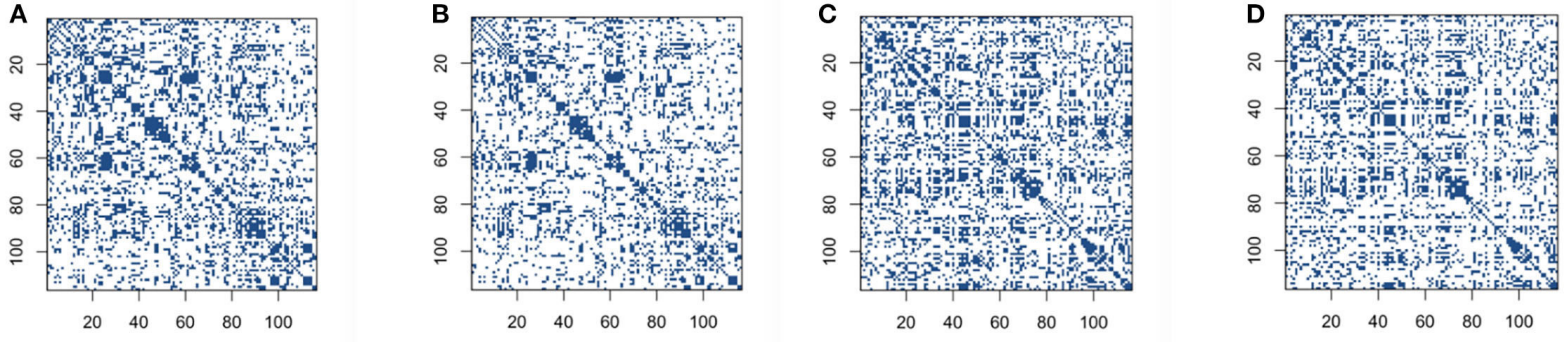
Connectivity selection results for the control and AD groups from correlation with thresholding: **(A)** control group based on original blood oxygen level dependent (BOLD) signals; **(B)** control group based on low-rank matrix; **(C)** Alzheimer's disease (AD) group based on original BOLD signals; **(D)** AD group based on low-rank matrix.

We first conduct correlation based on both the original BOLD signals and low-rank matrices. We set the reasonable hard threshold value 0.4 for correlation for both cases, in order to derive the sparse connectivity selection results. The connectivity selection results for correlation are shown in Figure 2. The dark blue dots represent the non-zero elements of connectivity matrices and white dots mean entries with zero values. From Figure 2, we can see that the patterns of connectivity matrices based on both the original BOLD signals and low-rank matrices for either the control group subjects or the AD group subjects are quite similar. Though we can also see that for both groups, the correlation matrices based on low-rank matrices after employing thresholding values are slightly sparser than the ones based on original BOLD signals.

The partial correlation based on both original BOLD signals and low-rank matrices are conducted. We set the reasonable hard threshold value 0.8 for partial correlation based on original data matrices, and 0.2 for partial correlation based on low-rank matrices. The results' graphs are omitted to conserve space. Graphical Lasso is also conducted to obtain the estimated sparse precision matrix automatically due to the method. In our study, the value ρ in (2) is chosen as 0.1 in order to get the reasonable sparsity, and also to achieve the comparable sparsity with other methods for construction. The results' graphs are again omitted to conserve space.

**Figure 3:**
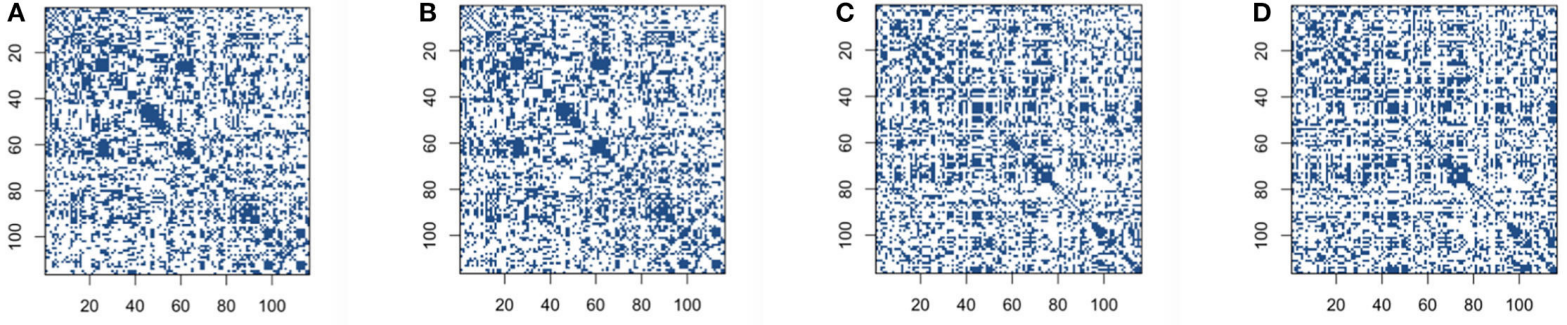
Connectivity selection results for control and Alzheimer's disease (AD) group from sparse covariance estimation method: **(A)** control group based on original blood oxygen level dependent (BOLD) signals; **(B)** control group based on low-rank matrix; **(C)** AD group based on original BOLD signals; **(D)** AD group based on low-rank matrix.

We then build the functional connectivity matrices using the recently proposed novel concentration inequality-based estimation method of sparse covariance matrices. We choose false positive rate α=0.35 in order to get the reasonable sparsity for the estimation of sparse covariance matrices, and also to achieve the comparable sparsity with other methods for construction. The sparsity of connectivity matrices for the concentration inequality-based estimation method is around 64%. Our sparse estimators ${\hat{\Sigma }}^{sp}$ results using concentration inequality-based method for both original BOLD signals and low-rank matrices are shown in Figure 3.

The comparison of same panels in Figures 2A, 3A shows that the patterns for the significant pairs are similar based on thresholding and sparse covariance estimation method for both control and AD group subjects. This indicates that the recently proposed novel concentration inequality-based estimation method performs well in terms of the estimation of sparse covariance matrices for both cases based on original BOLD signals and low-rank matrices. Therefore, we declare that this method supplies us with a novel and efficient way for constructing functional connectivity matrices. Through controlling the parameter false positive rate α in this method, we can achieve the desired sparsity for our estimation of sparse covariance matrices.

Furthermore, as can be seen in Figure 3, the patterns of connectivity matrices based on both the original BOLD signals and low-rank matrices for both groups are still quite similar, which indicates that *L* + *S* decomposition method can identify the essential common features while still retaining most of features for concentration inequality-based estimation method.

### 5.3. Comparison and Discussion

From the previous outputs and analyses, we have seen that for each single subject and for each method we employ for construction, we already derive connectivity results based on both original BOLD signals and low-rank matrices. Subsequently we conduct two sample *T* statistical test for each single ROI connection, in order to reveal and identify the underlying group differences between the control group and AD group. In our study, we have 6,670 total connections [(116 × (116 − 1)/2)]. Two sample *T* statistical test is implemented to each single connection *c*_*p,q*_ of *p*th ROI and *q*th ROI. Then the *p*-value matrix with 116 × 116 dimension for each single method we use is derived based on both original BOLD signals and low-rank matrices. The *p*-value matrices supply us the significant connection locations for differentiating the control group and AD group, which can intensely contribute to uncovering underlying neurological processes associated with AD for clinical use. Once we get the *p*-value matrix, we need a reasonable significant level α. The adjusted *p*-values are normally used in multiple comparisons (Wright, 1992). But in our study, we do not employ adjusted *p*-values. The reason is that the overlap rates for significant connection locations based on original BOLD signals and low-rank matrices start getting stable from threshold value 0.04 onwards, which we will illustrate in more detail later. Hence, we choose 0.05 as the significant level.

After getting the *p*-value matrices, we first focus on the quantities of significant ROI connections out of 6,670 total connections. That is to say, we explore how many connections can be considered significant in terms of distinguishing the differences between the normal group and AD group. We define and calculate the percentage of significant connections and compare the results based on both original BOLD signals and low-rank matrices. Under significant level 0.05, we define the percentage of significant connections of *p*-value matrix as the number of elements smaller than 0.05 divided by 6,670. Then we have the following comparison results based on both original BOLD signals and low-rank matrices for all the methods we employed in our study, as shown in Table 1.

**Table 1:** Percentage comparisons of significant connections.

**Methods**	**Original BOLD signals**	**Low rank matrix**
Correlation	6.3868	6.3718
Sparse correlation	5.7121	5.9970
Partial correlation	3.2684	4.5577
Sparse partial correlation	3.6732	4.7826
Glasso precision	4.6927	4.9775
Glasso partial correlation	4.7077	4.7077
Sparse covariance estimation	5.8321	5.9220

From Table 1, we can see that except the correlation method and Glasso partial correlation method, all the other methods for construction based on low-rank matrices have higher percentage of significant connections than based on the original BOLD signals. This suggests that the former behaves better than based on the original BOLD signals, in the sense that it can reveal and identify more significant ROI connections between the control group and AD group. Therefore, we verify performing *L* + *S* matrix decomposition can help us achieve more differentiable results for functional brain connectivity. Furthermore, we can also see that the recently proposed concentration inequality-based method performs better overall compared with other methods.

Subsequently, we demonstrate the first 10 most significant pairs of ROIs with first ten smallest *p*-value for both sparse correlation method and concentration inequality-based estimation, both based on low-rank matrices, as shown in Tables 2, 3, which give us the most important ROI pairs for distinguishing normal subject and AD subject we should pay more attention to in clinical use. We can also see that the connection between left hippocampus region and left cerebellum 7 region is the most significant ROI connection for differentiating the normal group and AD group for AD, with *p*-value smaller than 0.0005. Researchers have shown that the cerebellum has a strong role in higher cognitive functions which include memory processes, and possibly serves long-term memory encoding and information storage (Filippini et al., 2009). It has been also demonstrated that AD patients showed abnormal hippocampal connectivity during resting state (Wang et al., 2006). Other research has illustrated that the connectivity between hippocampus and cerebellum area is significantly different for the control group and AD group (Allen et al., 2007). Therefore, our finding here is consistent with existing literatures' findings.

**Table 2:** The first 10 most significant pairs for sparse correlation based on low-rank matrices.

**Pair**	**Region 1**	**Classification**	**Region 2**	**Classification**	**p-value**
1	L.HIP	Limbic lobe	L.CER7	Cerebellum	0.00010
2	L.CER45	Cerebellum	VER7	Vermis	0.00045
3	L.MFG	Frontal	L.IFGtriang	Frontal	0.00049
4	R.SFGdor	Frontal	L.IFGtriang	Frontal	0.00062
5	R.ANG	Parietal	VER8	Vermis	0.00096
6	R.INS	Insula	R.SMG	Parietal	0.00101
7	R.ORBsupmed	Frontal	L.ITG	Temporal	0.00104
8	L.SMA	Frontal	R.CUN	Occipital	0.00110
9	L.IFGoperc	Frontal	R.IFGtriang	Frontal	0.00113
10	L.SFGdor	Frontal	VER9	Vermis	0.00123

**Table 3:** The first 10 most significant pairs for concentration inequality-based estimation method based on low-rank matrices.

**Pair**	**Region 1**	**Classification**	**Region 2**	**Classification**	**p-value**
1	L.HIP	Limbic lobe	L.CER7	Cerebellum	0.00015
2	L.MFG	Frontal	L.IFGtriang	Frontal	0.00020
3	R.REC	Frontal	R.SOG	Occipital	0.00028
4	L.SMA	Frontal	R.CUN	Occipital	0.00033
5	L.CAU	Corpus striatum	L.TPOsup	Limbic	0.00062
6	R.SFGdor	Frontal	L.IFGtriang	Frontal	0.00085
7	L.ITG	Temporal	VER6	Vermis	0.00101
8	R.ORBsupmed	Frontal	R.ITG	Temporal	0.00121
9	L.OLF	Frontal	L.CER6	Cerebellum	0.00140
10	R.ANG	Parietal	VER8	Vermis	0.00163

**Figure 4:**
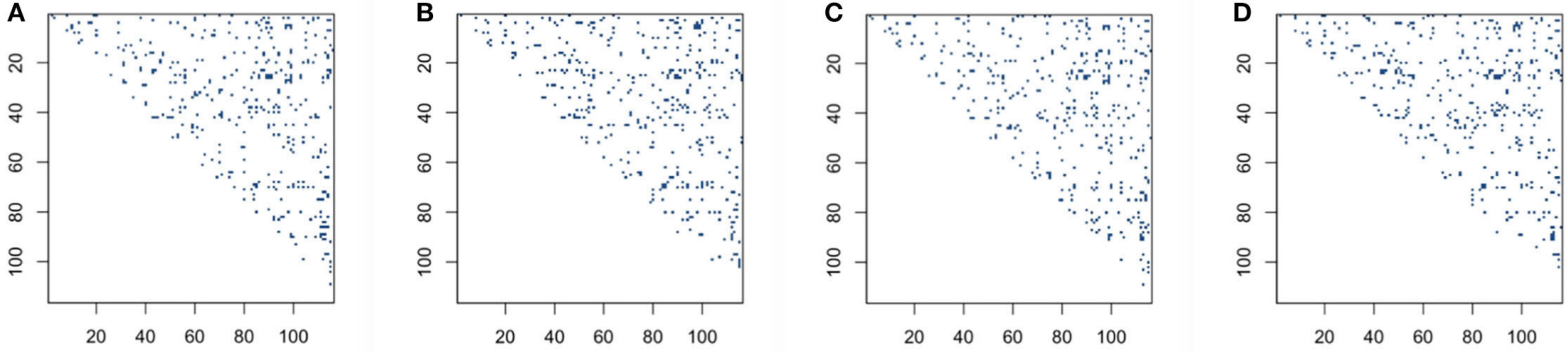
Significant connection location detecting for sparse correlation and sparse covariance estimation method: **(A)** sparse correlation based on original blood oxygen level dependent (BOLD) signals; **(B)** sparse correlation based on low-rank matrices; **(C)** sparse covariance estimation based on original BOLD signals; **(D)** sparse covariance estimation based on low-rank matrices.

We then explore whether performing *L* + *S* matrix decomposition when studying functional brain connectivity can keep some level of consistency compared with when using original data matrices. In order to see qualitatively the distribution comparison of those significant connection locations based on original BOLD signals and low-rank matrices, we draw significant connection location graphs for sparse correlation method and sparse covariance estimation method, which are shown in Figure 4. The dark blue dots represent the significant connection locations, or the locations for the *p* < 0.05 in the *p*-value matrices. As shown in Figure 4, the significant connection locations detected have visually similar distribution, which indicates decent overlap, for both based on original BOLD signals and low-rank matrices, for the sparse correlation method and also for the sparse covariance estimation method.

Furthermore, in order to go a step further to quantitatively check significant connection location distribution to see the overlap status for both original BOLD signals and low-rank matrices, we define and calculate the overlap rate. We denote *p*^*original*^ as the *p*-value matrix based on original BOLD signals, and *p*^*lowrank*^ as the *p*-value matrix based on low-rank matrices. Here, we only focus on the upper triangle of the symmetric *p*-value matrices. For the counterpart elements $p_{\left(i,j\right)}^{original}$ and $p_{\left(i,j\right)}^{lowrank}$, where *i* < *j*, we denote *n*_1_ as the number of elements of upper triangle which satisfy $0.05>p_{\left(i,j\right)}^{original}>p_{\left(i,j\right)}^{lowrank}$, *n*_2_ as the number of elements of upper triangle which satisfy $p_{\left(i,j\right)}^{original}<p_{\left(i,j\right)}^{lowrank}<0.05$, and $n_{non-zero}^{original}$ the number of non-zero of the upper triangle of *p*-value matrix based on original data. Then we define overlap rate as


$$\begin{array}{l} \left(n_{1}+n_{2}\right)/n_{non-zero}^{original}. \end{array}$$

The results for overlap rate based on different methods we utilize in our study are shown in Table 4. From Table 4, we can see that the overlap rates for correlation method, sparse correlation method, and sparse covariance estimation method are relatively large enough, while the other methods are not. Furthermore, the concentration inequality-based estimation has slightly higher overlap rate than sparse correlation method. These findings indicate that building brain connectivity based on the low-rank matrices when using the correlation method, the sparse correlation method, and the sparse covariance estimation method can achieve decent level of consistency, in the sense of the overlap status compared with the outputs based on original BOLD signals. Furthermore, the concentration inequality-based estimation has better consistency result than sparse correlation. Hence, we verify that performing *L* + *S* matrix decomposition when we study functional brain connectivity can keep decent level of consistency when using original BOLD signals.

**Table 4:** Overlap rate (%).

**Methods**	**Overlap rate**
Correlation	77.93
Sparse correlation	58.53
Partial correlation	4.13
Sparse partial correlation	5.31
Glasso precision	6.39
Glasso partial correlation	5.41
Sparse covariance estimation	59.90

**Figure 5:**
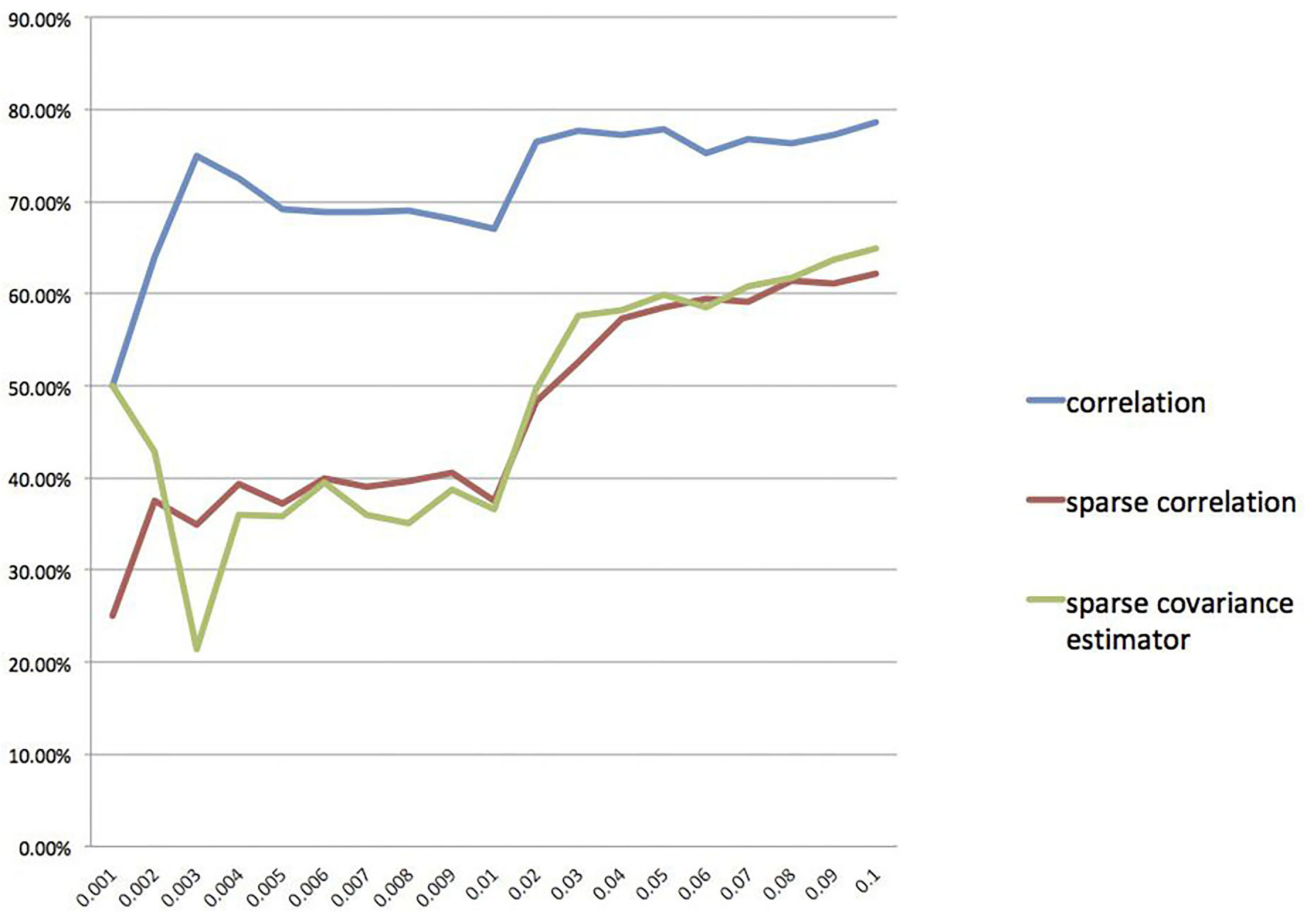
Overlap rate for different threshold values.

Furthermore, we also calculate the overlap rates under different threshold values for the correlation method, the sparse correlation method, and the sparse covariance estimation method. We draw a line chart for overlap rate to better demonstrate the results, as shown in Figure 5. We can see that the overlap rates start getting more stable from threshold value 0.04 onwards.

In order to verify if performing *L* + *S* matrix decomposition can achieve more stable results for constructing functional connectivity matrices, we implement bootstrapping for 50 times, and each time we sample 33 subjects out of 33 subjects in the control group and 24 subjects out of 24 subjects in the AD group, both with replacement. For each resampling, we conduct two sample *T* statistical tests to derive the *p*-value matrices based on both original BOLD signals and low-rank matrices. We apply this process to all the methods we employ for construction. As we stated above, for each *p*-value matrix we have a percentage of significant connections. Then for 50 times bootstrapping, we have 50 percentages of significant connections. Thus, we calculate the variance for percentages of significant connections, and the results are demonstrated in Table 5. As seen in Table 5, the variances of percentages of significant connections based on low-rank matrices are all smaller than those based on original BOLD signals for all the methods we employ for construction in our study. This result indicates that performing *L* + *S* matrix decomposition can achieve more stable results when constructing functional connectivity matrices.

**Table 5:** Variance comparisons of percentages of significant connections for 50 times bootstrapping (unit: ×10^−4^).

**Methods**	**Original BOLD signals**	**Low-rank matrix**
Correlation	5.6443	4.7270
Sparse correlation	5.6554	5.0379
Partial correlation	3.6149	3.4020
Sparse partial correlation	4.4523	3.6015
Glasso precision	4.0707	3.8987
Glasso partial correlation	3.0966	3.0435
Sparse covariance estimation	5.1630	4.0835

## 6. Conclusion and Discussion

In this paper, we propose a pipeline architecture that enables us to provide robust and stable functional brain connectivity, and increase the detectability of group differences. In particular, an *L* + *S* matrix decomposition technique is adapted to decompose the ADNI data, where the low-rank matrix *L* recovers the essential common features from ROIs, and the sparse matrix *S* catches the sparse individual variability and potential outliers. We apply our construction methods based on low-rank matrices from decomposition and compare the results with those based on original BOLD signals. We discover that the methods for building connectivity matrices based on low-rank matrices behave better than based on original BOLD signals, in the sense that the methods we employ for constructing connectivity matrices based on low-rank matrices can reveal and identify more significant ROI connections for group differences. Hence, this suggests that when we study group difference for functional brain connectivity, performing *L* + *S* matrix decomposition can achieve more differentiable results, which can contribute to uncovering underlying neurological processes associated with the disease for clinical use. We also find that the recently proposed concentration inequality-based method performs better overall compared with correlation, partial correlation, and Graphical Lasso method for connectivity construction. We verify that this method supplies us a novel and efficient way to explore functional brain connectivity. The pipeline architecture that we propose can be generalized to other datasets potentially to achieve robust and stable results.

Furthermore, we obtain the first ten most significant connections for differentiating group differences for AD. Among them, the left hippocampus region and the left cerebellum seven region is the most significant one, with *p*-value smaller than 0.0005, which is consistent with existing literatures' findings. Moreover, through bootstrapping, we verify that performing *L* + *S* matrix decomposition can achieve more stable results for constructing functional brain connectivity matrices.

## Data Availability Statement

Publicly available datasets were analyzed in this study. This data can be found here: http://adni.loni.usc.edu.

## Author Contributions

WT and FF are responsible for the majority of the writing and analysis. LK, BJ, and CH are responsible for the analysis and study design. DC is responsible for the analysis and writing–editing. All authors contributed to the article and approved the submitted version.

## Conflict of Interest

The authors declare that the research was conducted in the absence of any commercial or financial relationships that could be construed as a potential conflict of interest.

## Publisher's Note

All claims expressed in this article are solely those of the authors and do not necessarily represent those of their affiliated organizations, or those of the publisher, the editors and the reviewers. Any product that may be evaluated in this article, or claim that may be made by its manufacturer, is not guaranteed or endorsed by the publisher.

## Footnotes

^1^Data used in preparation of this paper were obtained from the Alzheimer's Disease Neuroimaging Initiative (ADNI) database (adni.loni.ucla.edu). As such, the investigators within the ADNI contributed to the design and implementation of ADNI and/or provided data but did not participate in the analysis or writing of this report. A complete listing of ADNI investigators can be found at: http://adni.loni.usc.edu/wp-content/uploads/how_to_apply/ADNI_Acknowledgement_List.pdf.
